# Prevention practices of hepatitis B virus and its associated factors among barbers in East Gojjam Zone, Northwest Ethiopia

**DOI:** 10.3389/fpubh.2025.1445543

**Published:** 2025-04-07

**Authors:** Baye Tsegaye Amlak, Benalfew Lake Mengistie, Seblework Abraham Teshale

**Affiliations:** ^1^Department of Nursing, College of Health Sciences, Debre Markos University, Debre Markos, Ethiopia; ^2^Department of Nursing, Yejube Primary Hospital, Yejube, Ethiopia

**Keywords:** prevention practices, factors, hepatitis B virus, barber, Ethiopia

## Abstract

**Introduction:**

Barber-related infections, including hepatitis B virus (HBV), continue to be a major cause of illness and death. Numerous beauticians use razors and scissors on multiple customers without adequately sanitizing these tools. There is a lack of published research on the prevention practices and associated factors of hepatitis B virus infection among barbers in Ethiopia. Therefore, this study aimed to assess the practice and associated factors of hepatitis B virus infection among barbers.

**Method:**

A cross-sectional study was carried out involving 411 barbers selected through simple random sampling. Data collection was performed using an interviewer-administered questionnaire and an observational checklist. The collected data were first cleaned and entered into EpiData version 4.6 and then exported to SPSS version 25 for analysis. Model fitness was assessed using the Hosmer–Lemeshow test, and multicollinearity was evaluated with the variance inflation factor. A binary logistic regression model was employed for the analysis. To address confounding factors, explanatory variables with a *p*-value of less than 0.25 in the bivariable logistic regression were included in the multivariable logistic regression analysis. Factors with a *p*-value of less than 0.05 in the multivariable analysis were considered statistically significant.

**Results:**

Among the 411 participants, 328 (79.8, 95% CI: 75.6–83.6%) exhibited unsafe hepatitis B virus infection prevention practices. Unsafe practices were significantly associated with barbers who could not read or write (AOR 3.75, 95% CI: 1.39–10.12); primary and secondary education (AOR 3.44, 95% CI: 1.89–6.27) compared to those with college education and above; not using ultraviolet sterilizers (AOR 2.85, 95% CI: 1.30–6.27); insufficient knowledge (AOR 4.23, 95% CI: 2.13–8.40); unfavorable attitudes toward infection control (AOR 2.40, 95% CI: 1.34–4.31); and working hours of less than 8 h (AOR 0.27, 95% CI: 0.15–0.50).

**Conclusion:**

Nearly four-fifths of barbers exhibited unsafe practices in preventing hepatitis B virus infection. Low education levels, not utilizing UV sterilizers, lack of knowledge, working fewer hours, and negative attitudes toward infection prevention were all strongly associated with unsafe practices in the prevention of hepatitis B virus among barbers. Consequently, these findings underscore the need for targeted educational programs, improved access to sterilization tools, and policy changes to promote safer practices.

## Introduction

Hepatitis B virus (HBV) is a serious and potentially life-threatening infection that affects the liver (1). It is a major global public health problem (1).The highest burden of hepatitis B infection is observed in the WHO Western Pacific Region and the WHO Africa Region, with approximately 116 million and 81 million chronically infected people, respectively (2).

Barber-related infections, including hepatitis B virus (HBV), remain a significant cause of illness and death, particularly in emerging and impoverished countries. These challenges are worsened by factors such as poverty and poor sanitation (3, 4). The prevalence of HBV among barbers in Sudan is 10.1% (5).

In regions where unsafe hair-cutting practices are prevalent, there is an increased risk of HBV transmission among various populations, particularly those who frequently visit barbershops. It has been highlighted that individuals who are living in areas with high HBV prevalence are particularly vulnerable as regular visits to barbershops with inadequate infection control measures put them at heightened risk (6).

Unsafe hair-cutting practices are increasingly recognized as a significant risk factor for the transmission of HBV, especially in settings where hygiene standards are not adequately maintained. Studies have shown that barbers and hairdressers often use sharp tools, such as razors and clippers, which may come into contact with blood. If these tools are not properly sterilized, there is a high risk of transmitting HBV through minor cuts, nicks, or shared equipment (7).

Despite the significant occupational risks faced by barbers, those in developing countries often have limited knowledge about HBV (8–10). A study in Pakistan showed only moderate awareness among barbers about the various modes of transmission of hepatitis (10).

Shaving is a widely practiced cultural activity in barbershops and roadside barber setups across much of Africa. This common practice can potentially facilitate the spread of HBV (11). Compared to the human immunodeficiency virus (HIV), HBV is 50 to 100 times more contagious (12).

Despite their vital role in the community and the continuous support, many barbers do not follow proper and hygienic haircutting practices (13). This activity can promote the spread of certain viruses, thereby contributing to a higher incidence of infectious diseases in many developing countries (14).

Barbers engage in cutting different types of hair, shaving, and trimming beards as part of their work. They are especially vulnerable to infections due to frequent exposure to wounds and scratches caused by sharp tools (11). Hair salons can act as centers for the spread of various contagious diseases, making them potentially hazardous environments. The risk increases significantly if equipment is not thoroughly decontaminated between clients (15).

Many barbers use razors and scissors on multiple customers without properly cleaning them, reflecting a lack of awareness about the risks of spreading germs and viral hepatitis (16). Instruments used in barbershops include trimmers, scissors, hair clippers, razors, blades, shavers, capes, and scarves. Proper sterilization or disinfection of these instruments is important to prevent the transmission of health hazards, including HBV (17).

In many barbershops, the risk of infection is high due to the use of equipment that has not been properly cleaned or decontaminated. This risk extends from patrons to other customers and is further exacerbated if the barber has unprotected cuts or bruises (14). Many individuals use barbershop services in their communities without being aware of these risks. As a result, barbers’ workplaces and activities may become hidden sources of community transmission of communicable diseases (14, 16).

Barbers often have inadequate practices for preventing HBV infections. A lack of awareness about health risks leads to poor habits, insufficient decontamination, and ineffective preventive measures in the barbering industry, creating an environment that facilitates the transmission of HBV (17, 18).

Key risk factors for the transmission of HBV include sharing razors, inadequate sterilization and decontamination of equipment, use of ineffective or questionable cleaning solutions, barbers lacking proper personal protective equipment, and careless handling of sharp objects (11, 14, 16, 17, 19, 20). Barbers in Ethiopia have minimal training and experience in managing the biological hazards associated with their profession (16).

In low-resource countries, most barbers are not vaccinated against HBV, putting them at risk of contracting the virus through unintentional contact with a customer’s blood or bodily fluids while cutting or styling hair (21).

National and municipal health departments, public health agencies, and professionals are highlighting the serious implications of infectious diseases such as hepatitis B associated with this profession through national campaigns and initiatives, including print and electronic media. Despite these efforts, standards in hairdressing practices remain insufficiently high (20).

Despite its significant impact, severity, and adverse consequences, there are limited published studies on the practices and associated factors related to the prevention of HBV among barbers, specifically in the East Gojjam Zone. Therefore, this study aimed to determine the level prevention practice of HBV and identify factors associated with it in Northwest Ethiopia.

## Materials and methods

### Study design

Cross-sectional study was employed.

### Study area and period

The study was carried out in the East Gojjam Zone, located in the Amhara Regional State of Northwest Ethiopia, with its central city being Debre Markos. Debre Markos is approximately 299 kilometers from Addis Ababa, the capital city of Ethiopia, and approximately 268 kilometers from Bahir Dar, the capital of the Amhara Regional State. According to the 2014 Census by the Central Statistical Agency of Ethiopia, the zone has a population of 2,451,959 total population (1,199,952 males and 1,252,006 females) (22). Within the zone, there are 9 city administrations and a total of 836 barbers. The study took place from 1 April 2023 to 15 June 2023.

### Source population

All barbers who were working in East Gojjam Zone city administrations.

### Study population

Barbers who were working in randomly selected cities in East Gojjam city administrations.

#### Inclusion criteria

All barbers who were working in East Gojjam Zone city administrations.

#### Exclusion criteria

Participants whose barbershops were closed during the data collection period were excluded from the study.

### Sample size determination

The sample size was calculated to assess both the level of prevention practices toward HBV and its associated factors. The calculation for the level of practice was based on the single population proportion formula. As the practice of HBV infection prevention and its associated factors had not been previously studied in Ethiopia, an assumption was made that 50% of barbers engaged in unsafe practices (23). With a confidence level of 95%, a margin of error of 5%, and considering a 10% non-response rate, the sample size was determined accordingly. The sample size was calculated by using the statistical formula for the practice level:


$$n=\left(z_{\alpha 2}\;p\;\left(1-p\right)\right)/d^{2}$$

where *n* = desired sample size

*Z*_*a/*2_ = *Z* score at d = 95% confidence level = 1.96,

*p* = 50% = 0.5.

D = margin of error = 0.05.


$$n=\left(z_{\alpha 2}\;p\;\left(1-p\right)\right)/d^{2}$$

*n* = (1.96)^2^ (0.5) (0.5)/(0.05)^2^ n = 384

A sample size of 384 was used and after adding a non-response rate of 10%, the final sample size was 423. Sample size for associated factors was calculated (Table 1).

**Table 1:** Sample size calculation for the associated factors toward HBV prevention practice among Barbers in Northwest Ethiopia.

Factors	CI	Power	Ratio	% Outcome in unexposed	% Outcome in exposed	Sample size	After adding 10% non-response rate	Reference
Working hours	95%	80%	1	92.74%	77.18%	190	209	(16)
Residence	95%	80%	1	75.27%	93.97%	136	150	(39)

The largest sample size was found on using single population proportion formula, which was 423.Therefore, the final sample size was 423.

### Sampling technique and procedure

**Figure 1:**
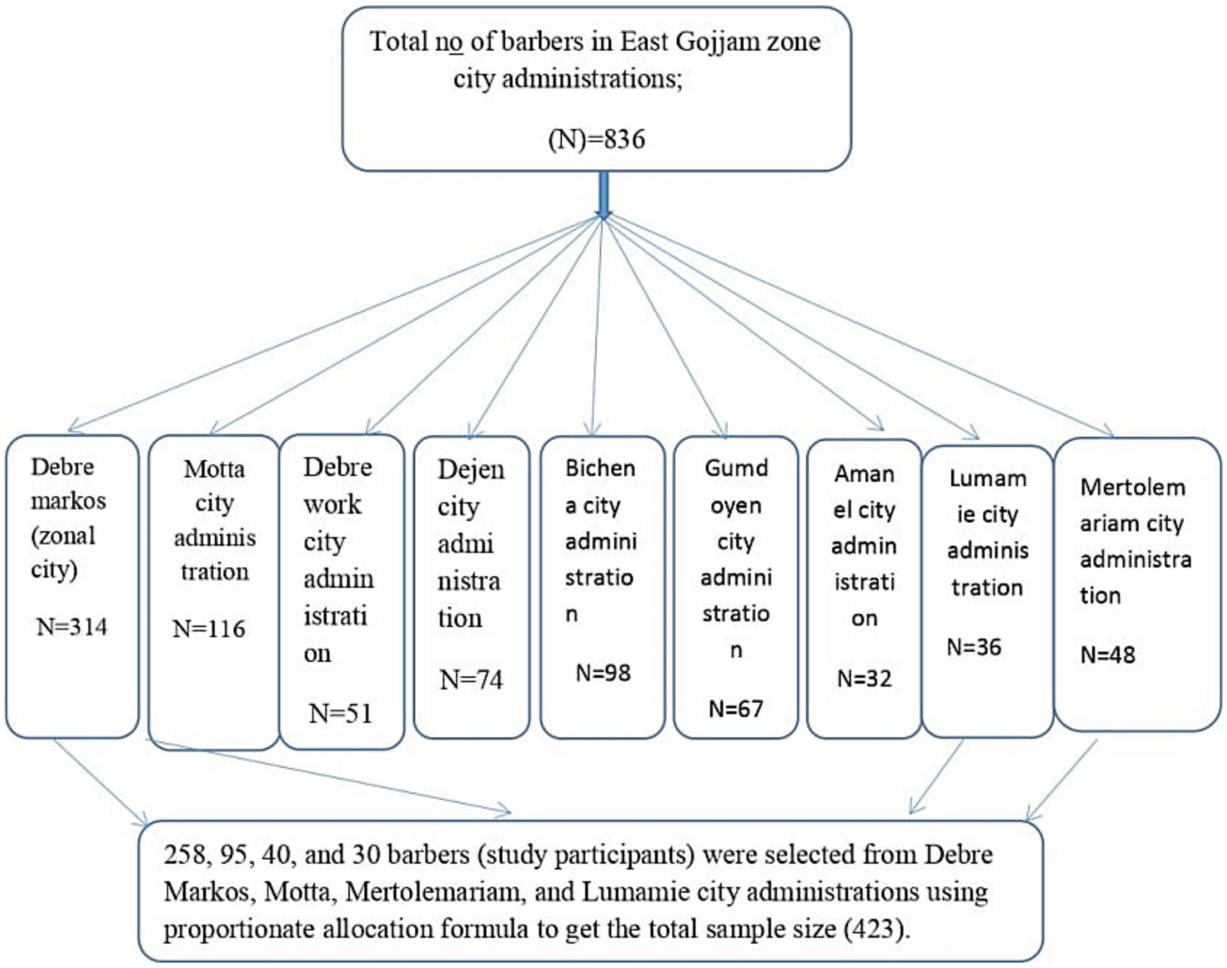
Sampling procedure for practice and associated factors toward prevention of hepatitis B virus among barbers in East Gojjam Zone city administrations, Northwest Ethiopia, 2023.

Among the nine city administrations, four of them, namely, Debre Markos, Motta, Mertolemariam, and Lumamie, were randomly selected using a lottery method. The proportionate allocation formula was used to distribute the sample size across the selected city administrations. Within each barbershop, one barber was randomly selected to participate in the study using a lottery method (Figure 1).

### Variables

#### Dependent variable

Barbers prevention practices regarding hepatitis B virus prevention (safe vs. unsafe practices).

#### Independent variables

The independent variables of this study included sociodemographic factors, such as age, sex, educational level, marital status, religion, income, working hours, and work experience, the presence of an ultraviolet sterilizer, and the participants’ knowledge and attitude levels.

### Operational definitions

#### Adequate knowledge

Fourteen knowledge-related questions were used to measure the level of knowledge. These questions were structured as Yes or No, with participants earning a score of ‘1’ for a correct answer and ‘0’ for an incorrect one. The total possible score ranged from 0 to 14. Participants who correctly answered more than 50% (7 out of 14) of the questions were categorized as having adequate knowledge (24, 25).

#### Inadequate knowledge

Respondents who correctly answered 50% or fewer of the knowledge questions correctly were considered to have inadequate knowledge (24, 25).

#### Favorable attitude

Attitude was assessed using 10 Likert-type questions, with responses ranging from 1 (strongly disagree) to 5 (strongly agree). The maximum possible score was 50, whereas the minimum score was 10. Respondents who correctly answered more than the mean score of 10 attitude questions of the respondents after calculating the mean using SPSS were considered to have a favorable attitude (26).

#### Unfavorable attitude

Respondents who correctly answered at or below the mean score of attitude questions of the respondents after calculating the mean using SPSS were considered to have an unfavorable attitude (26).

#### Safe practice

The practice level of the respondents was evaluated using 12 Yes or No questions related to practice. Participants received a score of 1 for each correct answer and 0 for each incorrect or unanswered question. The total possible score ranged from 0 to 12. Respondents who correctly answered more than 50% (more than six questions) of the practice questions were classified as having safe practices (24–26).

#### Unsafe practice

Respondents who answered 50% or fewer of the practice questions correctly were classified as having unsafe practices (24–26).

NO answer: in the knowledge, and practice questions No refers to the combined response of “No” and “Do not know.”

#### Clean

A barbershop is considered clean if there is no visible dust or dirt on the floor or walls, and if the instruments are shiny, and properly organized (27).

#### Attractive

A barbershop is considered attractive if it draws interest through high-quality products, product displays, and a good location or accessibility (28).

#### Ventilated

A typical situation where outdoor air is exchanged with indoor air through a single window opening (29).

### Data collection tools and procedure

An interviewer-administered questionnaire was used to gather data on respondents’ socio-demographic characteristics (such as age, sex, educational status, and work experience), their practices regarding HBV prevention (using 12 questions), and their knowledge (using 14 Yes or No questions) and attitudes (using 10 Likert-scale questions) toward HBV transmission and prevention methods. Additionally, an observational checklist was used to collect data.

Data collection was carried out by four BSC nurses under the supervision of two BSC supervisors.

### Data quality control

Training was provided to both data collectors and supervisors. A pretest was conducted on a 5% sample of the population in Basoliben Woreda 1 week prior to the actual data collection. This pretest aimed to assess the clarity of the data collection tools. Data collectors were trained to reduce ambiguity when respondents required assistance and to enhance the clarity of the information gathered. After data collection, each questionnaire was reviewed for errors and completeness. The collected data were properly handled and stored until analysis. The reliability of the questionnaires was checked using Cronbach alpha with the value of 0.741. This was very important to identify the clarity of the questions in the questionnaire, the time taken to complete the questions, and also, modification has been done.

### Data processing and analysis

First, the data were checked for completeness and consistency. Then, it was coded and entered into EpiData version 4.6. After that, the data were exported to SPSS version 25 for analysis. Model fit was assessed using the Hosmer–Lemeshow test, which indicated good fit with a *p*-value of 0.48. Multicollinearity was checked using variance inflation factors, with a maximum value of 1.75.

A binary logistic regression model was used to identify factors associated with the status of practices among barbers. This was done by calculating the odds ratio and *p*-value.

Explanatory variables with a *p*-value less than 0.25 in the bivariable logistic regression were entered into the final multivariable logistic regression analysis to control for possible confounding and to perform further analysis. Variables with a *p*-value less than 0.05 were considered significantly associated with the dependent variable.

Descriptive analysis using frequencies, proportions, and graphs was performed to describe the number and percentage of sociodemographic characteristics and other variables in the sample.

### Ethical considerations

Ethical clearance was obtained from the Institutional Research Ethics Review Board (IRERC) of Debre Markos University. The IRERC had reviewed the study protocol and approved it. The approval number provided by IRERC was R/C/S/D/102/01/23. Written informed consent was used. The data were not disclosed to any person other than the principal investigator. Confidentiality of the information was maintained throughout the study. An explanation of the objective of the study was given to the study participants. Written informed consent was used. In addition, affirmation that they are free to withdraw consent and to discontinue participation was made. To ensure confidentiality of the patients’ information, their names and address of the patients were not recorded during the data collection. The investigator used the collected data only to answer the stated objectives. This study was conducted in accordance with the Declaration of Helsinki.

## Results

Out of 423 participants, 411 actually took part in the study, resulting in a response rate of 97.1%. Among these respondents, 335 (81.5%) were male. Approximately 39.9% of the participants were aged between 20 and 29 years, with a mean age of 31.69 years and a standard deviation of ±8.728. The majority, 380 (92.5%), were followers of the Orthodox Christian religion. Approximately 249 (60.6%) had less than 5 years of experience. Additionally, 322 (78.3%) of the participants worked greater than 8 h a day. Nearly three-fourths of the participants, 310 (75.4%), used UV sterilizers. Nearly two-thirds (63.0%) of the participants had a monthly income ranging from 3,000 to 5,000 Ethiopian birr (Table 2).

**Table 2:** Sociodemographic characteristics of the barbers in East Gojjam Zone, 2023 (*n* = 411).

Variable	Category	Frequency	Percent
Sex	Male	335	81.50
	Female	76	18.50
Age in years	15–20	29	7.10
	20–29	164	39.90
	30–39	134	32.60
	40–49	67	16.30
	≥50	17	4.10
Religion	Orthodox	380	92.50
	Muslim	25	6.10
	Protestant	6	1.50
Education	Unable to read and write	48	11.70
	Primary and secondary school	261	63.50
	College	95	23.10
	Degree and above	7	1.70
Marital	Single	169	41.10
	Married	234	56.90
	Divorced	7	1.70
	Widowed	1	0.20
Working hours	<8	89	21.70
	> = 8	322	78.30
Experience in years	<5	249	60.60
	5–10	103	25.10
	>10	59	14.40
Income in Ethiopian birr	<3,000	51	12.40
	3,000–5,000	259	63.00
	>5,000	101	24.60
Ultraviolet use	Yes	310	75.40
	No	101	24.60

### Knowledge of barbers about hepatitis B virus

Out of the total participants, 355 (86.4%) had inadequate knowledge, whereas 56 (13.6%) had adequate knowledge, based on the knowledge questions they answered according to the operational definitions provided above (Table 3).

**Table 3:** Knowledge of barbers about hepatitis B virus in East Gojjam Zone, 2023 (*n* = 411).

Knowledge	Level	Frequency	Percentage
Is hepatitis viral disease?	No	287	69.80
	Yes	124	30.20
Is hepatitis B transmitted by blood and body fluid?	No	338	82.20
	Yes	73	17.80
HBV transmitted by blades?	No	191	46.50
	Yes	220	53.50
HBV spread via sexual contact?	No	338	82.20
	Yes	73	17.80
Is jaundice one of the common symptom of HBV?	No	311	75.70
	Yes	100	24.30
HBV mostly affects liver?	No	218	53.00
	Yes	193	47.00
HBV can lead to cancer	No	368	89.50
	Yes	43	10.50
HBV can be transmitted from mother to child during pregnancy?	No	359	87.30
	Yes	52	12.70
HBV leads to lifelong infection?	No	315	76.60
	Yes	96	23.40
HBV has laboratory test?	No	211	51.30
	Yes	200	48.70
HBV has vaccine?	No	389	94.60
	Yes	22	5.40
HBV has post exposure prophylaxis?	No	385	93.70
	Yes	26	6.30
HBV has treatment?	No	180	43.80
	Yes	231	56.20
HBV infection can be prevented?	No	182	44.30
	Yes	229	55.70
Knowledge level	Adequate knowledge	56	13.6
	Inadequate knowledge	355	86.4

No = No plus do not know.

### Attitude of barbers about hepatitis B virus infection prevention

Approximately 228 (55.5%) of the barbers had a favorable attitude, whereas 183 (44.5%) had an unfavorable attitude. The attitudes of barbers were assessed through 10 questions, each rated from 1 to 5, with a total score range of 10 to 50. The mean score was used to classify attitudes: barbers who scored below or equal to the mean of 33.98 were considered to have an unfavorable attitude, whereas those who scored above the mean were regarded as having a favorable attitude (Table 4).

**Table 4:** Attitude of barbers on HBV infection prevention practices in East Gojjam Zone, 2023 (*n* = 411).

Questions	Scale	Frequency	Percent
Do you agree to be personally tested for HBV infection?	Strongly disagree	30	7.30
	Disagree	109	26.50
	Neutral	63	15.30
	Agree	198	48.20
	Strongly agree	11	2.70
Do you agree new blades to be used for each customer?	Strongly disagree	21	5.10
	Disagree	64	15.60
	Neutral	43	10.6
	Agree	230	56.00
	Strongly agree	53	12.90
Do you agree having tattoos can be a risk factor for HBV?	Strongly disagree	22	5.40
	Disagree	100	24.30
	Neutral	101	24.60
	Agree	165	40.10
	Strongly agree	23	5.60
Do you agree to take vaccination against HBV?	Strongly disagree	40	9.70
	Disagree	147	34.80
	Neutral	78	19.00
	Agree	139	33.80
	Strongly agree	7	1.70
Do you agree avoiding cuts can be preventive for HBV?	Strongly disagree	6	1.50
	Disagree	46	11.20
	Neutral	72	17.50
	Agree	221	53.80
	Strongly agree	66	16.10
Do you agree antiseptic use is necessary for barbershop?	Strongly disagree	3	0.70
	Disagree	30	7.30
	Neutral	71	17.30
	Agree	226	55.00
	Strongly agree	81	19.70
Do you believe using PPE is essential during shaving?	Strongly disagree	5	1.20
	Disagree	27	6.50
	Neutral	68	16.50
	Agree	223	54.30
	Strongly agree	88	21.40
Do you agree vaccination of family member is necessary?	Strongly disagree	38	9.20
	Disagree	83	20.20
	Neutral	95	23.11
	Agree	163	39.70
	Strongly agree	32	7.80
Do you agree avoiding intravenous drug abuse is preventive for HBV?	Strongly disagree	23	5.60
	Disagree	74	18.00
	Neutral	112	27.32
	Agree	173	42.10
	Strongly agree	29	7.10
Do you agree avoiding extramarital sex is protective against HBV?	Strongly disagree	11	2.70
	Disagree	61	14.80
	Neutral	113	27.50
	Agree	194	47.20
	Strongly agree	32	7.80
Attitude level	Favorable	228	55.5
	Unfavorable	183	45.5

### Prevention practice of barbers toward HBV infection prevention

**Figure 2:**
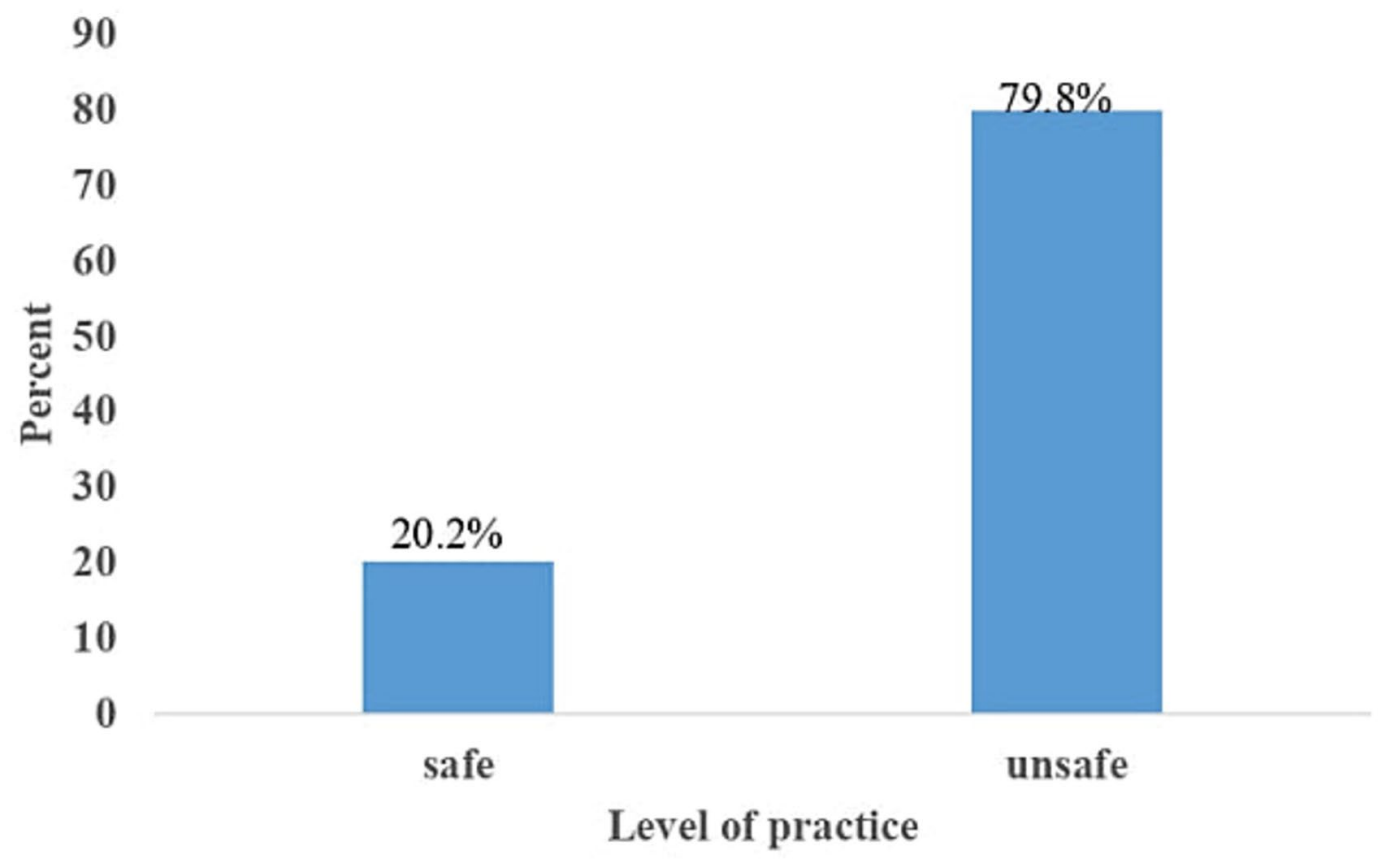
Hepatitis B virus prevention practice status among barbers in East Gojjam Zone city administrations, 2023.

In this study, 328 (79.8%) of the respondents had unsafe practices (95% CI [75.6–83.6%], Figure 2). Among the respondents, 99 (24.1%) washed their hands before and after cutting and shaving, 21 (5.1%) used gloves, 302 (73.5%) used a new blade for each customer, and 347 (84.4%) reused towels without sterilization. Additionally, 269 (65.5%) disinfected or sterilized instruments, 380 (92.5%) used razors for shaving, 103 (25.1%) changed combs, and 334 (81.3%) used an apron during shaving. Only 20 (4.9%) were screened for HBV, and 9 (2.2%) were vaccinated for HBV. Furthermore, 200 (48.7%) properly disposed of blades, and 216 (52.6%) managed cuts with antiseptics (Table 5).

**Table 5:** Barbers prevention practices toward HBV infection in East Gojjam Zone, 2023 (*n* = 411).

Practice questions	Category	Frequency	Percent
Do you wash your hands before each customer?	No	312	75.90
	Yes	99	24.10
Do you use glove for each client?	No	390	94.90
	Yes	21	5.10
Do you change blades after each client?	No	109	26.50
	Yes	302	73.50
Do you reuse towels without sterilization?	No	64	15.60
	Yes	347	84.40
Do you disinfect/sterilize instruments between customers?	No	142	34.50
	Yes	269	65.50
Do you use razor for shaving?	No	31	7.50
	Yes	380	92.50
Do you change comb for each client?	No	308	74.90
	Yes	103	25.10
Do you use apron during shaving?	No	77	18.70
	Yes	334	81.30
Have you ever undergone screening for HBV infection?	No	391	95.10
	Yes	20	4.90
Have you ever vaccinated against HBV infection?	No	402	97.80
	Yes	9	2.20
Do you properly dispose blades? (burial/burn)	No	211	51.30
	Yes	200	48.70
Do you manage cuts by antiseptics?	No	195	47.20
	Yes	216	52.60
Practice level	Safe	83	20.2
	Unsafe	328	79.8

### Observed results for barbers prevention practice toward HBV infection

#### From the observations

Approximately 284 (69.1%) of the barbershops were well ventilated, 222 (54.0%) of the barbershops were clean, 233 (56.7%) of the barbershops were considered attractive, and 212 (51.6%) had their own water supply. Nearly all participants (408) had an electricity supply; those without electricity used manual shavers.

#### Regarding practices

Approximately 359 (87.3%) of the respondents did not wash their hands. Almost all participants (408 or 99.3%) did not use gloves. Approximately 42 (10.3%) did not use a new blade for each customer. Approximately 130 (31.6%) reused towels without sterilization. Approximately 144 (35%) did not disinfect or sterilize instruments. Approximately 334 (81.3%) did not change combs; 388 (94.4%) used razors for shaving; approximately half of them, 223 (54.3%), did not properly dispose of blades; and 220 (53.5%) did not manage cuts with antiseptics.

### Factors associated with prevention practices of barbers toward HBV infection

In the bi-variable logistic regression analysis, the following variables were deemed eligible for inclusion in the multivariable logistic regression model based on a *p*-value of <0.25: educational status, income, working hours, knowledge, attitude of barbers, and ultraviolet sterilizer use.

In the multivariable logistic regression analysis, the predictor variables that were significantly associated with practice included educational status, working hours, knowledge, attitude of barbers, and the use of ultraviolet sterilizers.

This study found that barbers who could not read or write were nearly four times more likely to engage in unsafe practices (AOR 3.75, 95% CI [1.39–10.12]) than those with college education or higher. Additionally, barbers with primary and secondary school education were 3.4 times more likely to have unsafe practices (AOR 3.44, 95% CI [1.89–6.27]) than those with college education or higher.

Barbers who were using ultraviolet sterilizers were nearly three times more likely to have unsafe practices (AOR 2.85, 95% CI [1.30–6.27]) than those who did not use ultraviolet sterilizers.

This study revealed that barbers with inadequate knowledge were more than four times more likely to engage in unsafe practices (AOR 4.23, 95% CI [2.13–8.40]) than those with adequate knowledge. Additionally, barbers with an unfavorable attitude were more than two times as likely to have unsafe practices (AOR 2.40, 95% CI [1.34–4.31]) than those with a favorable attitude.

Furthermore, barbers who worked less than 8 h were 3.7 times less likely to practice unsafe methods (AOR 0.27, 95% CI [0.15–0.50]) than those working more than 8 h (Table 6).

**Table 6:** Bivariable and multi variable logistic regression results of factors affecting the barber’s prevention practices in East Gojjam Zone city administrations, 2023 (*n* = 411).

Variable	Category	Unsafe	Safe	COR(95%CI)	*p*-value	AOR (95%CI)	*p*-value
Educational status	Unable to write and read	41	7	3.20 (1.30–7.85)	0.011	**3.75 (1.39–10.12)**	**0.009**
	Primary and secondary	221	40	3.01 (1.78–5.11)	<0.001	**3.44 (1.89–6.27)**	**<0.001**
	College and above	65	36	1		1	
Working hours	<8 h	60	29	0.42 (0.25–0.71)	0.001	**0.27 (0.15–0.50)**	**<0.001**
	>8 h	268	54	1		1	
Income	<3,000	36	15	0.59 (0.27–1.29)	0.186	0.60 (0.24–1.48)	0.268
	3,000–5,000	211	48	1.09 (0.61–1.94)	0.782	1.09 (0.57–2.09)	0.797
	>5,000	81	20	1		1	
Ultraviolet use	No	92	9	3.21 (1.54–6.67)	0.002	**2.85 (1.30–6.27)**	**0.009**
	Yes	236	74	1		1	
Knowledge	Inadequate	296	59	3.76 (2.068–6.85)	<0.001	**4.23 (2.13–8.40)**	**<0.001**
	Adequate	32	24	1		1	
Attitude	Unfavorable	160	23	2.48 (1.47–4.21)	0.001	**2.40 (1.34–4.31)**	**0.003**
	Favorable	168	60	1		1	

Bold indicates statistical significant.

## Discussion

The aim of this study was to evaluate the prevention practices and associated factors related to the prevention of hepatitis B virus among barbers in East Gojjam, Northwest Ethiopia. In this study, 79.8% (95% CI: 75.6–83.6%) of the respondents had unsafe practice. This high prevalence of unsafe practices suggests the need for urgent intervention to improve awareness, training, and infection control measures in study setting.

This level of practice was higher than studies in Hawassa, Ethiopia 28.5% (30); Woldia, Ethiopia 59.5% (24); Mosul 58.33% (18); and Fiji (64.1%) (17). This discrepancy may be attributed to differences in socio-demographic factors, sample size, and economic status between regions (31). The sample size in the current study was relatively small, with 117 participants in Fiji and 60 participants in Mosul. In contrast, the sample size in Hawassa was larger, but there were differences in the items used to assess the practice level.

The findings of this study indicated that the level of unsafe practices was lower than those observed in studies conducted in Sudan (5), Yemen (32), Izmir (33), and Punjab (34). This discrepancy may be due to differences in access to healthcare infrastructure, resources, and technology among the countries, influenced by economic and other social factors. For example, barbers in more developed countries may have better access to sterilization equipment (e.g., ultraviolet sterilizers) and more disposable income to invest in these tools. In contrast, in developing countries like Ethiopia, economic constraints may limit access to such resources, leading to unsafe practices.

This study shows that barbers who did not use ultraviolet sterilizers (AOR 2.85, 95% CI: 1.30–6.27) were significantly associated with unsafe practices. This finding is supported by studies in Gondar, Ethiopia (16) and Hawassa, Ethiopia (30). This is because barbers who use ultraviolet sterilizers understand the significance of these devices. Ultraviolet sterilizers are highly effective in eliminating all types of microorganisms as they use light with a wavelength of at least 253.7 nanometers to disinfect bacteria, viruses, and other microorganisms (35, 36), thus preventing infections. However, if barbers do not use this equipment, they may transmit microorganisms from one customer to another, potentially even to themselves. Moreover, the use of proper sterilization equipment is essential for preventing HBV transmission in barbershops. Inadequate sterilization practices, including the failure to use ultraviolet sterilizers, have been documented as major risk factors for the spread of bloodborne infections (37). This highlights the need for policies to ensure barbershops have access to and use effective sterilization tools, particularly in resource-limited settings. Poor knowledge is likely to be associated with unfavorable attitudes (38).

In this study, Barbers who cannot read and write [AOR, 3.75, 95% CI (1.39–10.12)] and barbers who were at primary and secondary levels [AOR, 3.44, 95% CI (1.89–6.27)] were significantly associated with unsafe practice. This result was supported by studies in Gondar, Ethiopia (39), and Italy (40). This is because when barbers have inadequate knowledge about the causes, risk factors, and transmission routes of infections, they fail to properly clean, disinfect, and sterilize barbering instruments to eradicate microorganisms. Additionally, they may not use personal protective equipment to minimize cross-contamination for both their customers and themselves (41). As a result, infectious diseases can spread from person to person.

Moreover, it is obvious that improving the educational status of barbers may be a crucial intervention for bettering HBV prevention practices as education plays a crucial role in fostering the use of health-promoting habits. These results recommends the importance of ensuring that barbers receive adequate education and training, particularly in the areas of infection control and disease prevention.

This study found that barbers with inadequate knowledge (AOR 4.23, 95% CI: 2.13–8.40) and unfavorable attitudes toward infection control (AOR 2.40, 95% CI: 1.34–4.31) were more likely to be engaged in unsafe practices. This is due to a lack of awareness about HBV transmission and prevention methods can lead to negligent practices that increase the risk of infection (20). Similarly, unfavorable attitudes toward infection control may reflect a lack of motivation or perceived importance of adhering to safety procedures. These findings emphasize the need for organized training programs that maintain barbers with the necessary knowledge about HBV transmission, prevention strategies, fostering positive attitudes toward infection control, and the importance of infection control measures.

Barbers who worked fewer than 8 h were 3.7 (1/2.70) times less likely to practice unsafe methods (AOR 0.27, 95% CI 0.15–0.50) than those working more than 8 h, contrary to studies in Gondar (16) and Hawassa (30). Barbers who work less hours may have more time to devote to infection control practices, like properly sterilizing instruments and following hygiene guidelines. However, extended workdays may result in exhaustion, rushed processes, and a disregard for safety precautions.

## Conclusion

Barbers’ practices regarding the prevention of hepatitis B virus infection were found to be substandard. Unsafe practices were significantly associated with several factors, including lower education levels among barbers, non-use of ultraviolet sterilizers, inadequate knowledge, working less than 8 h, and having an unfavorable attitude toward hepatitis B infection prevention.

To improve the knowledge and practices of barbers, specific actions should be implemented, such as providing training on infection control. Barbers should use ultraviolet sterilizers and appropriate personal protective equipment to prevent the transmission of infectious diseases, including HBV. Qualitative studies could also be conducted to gain a deeper understanding of the poor attitudes and practices observed.

## Data Availability

The datasets presented in this study can be found in online repositories. The names of the repository/repositories and accession number(s) can be found in the article/supplementary material.
